# Cost-Effectiveness of Pembrolizumab Plus Chemotherapy Versus Pembrolizumab Monotherapy in Metastatic Non-Squamous and Squamous NSCLC Patients With PD-L1 Expression ≥ 50%

**DOI:** 10.3389/fphar.2021.803626

**Published:** 2022-01-10

**Authors:** Qiao Liu, Zhen Zhou, Xia Luo, Lidan Yi, Liubao Peng, Xiaomin Wan, Chongqing Tan, Xiaohui Zeng

**Affiliations:** ^1^ Department of Pharmacy, The Second Xiangya Hospital of Central South University, Changsha, China; ^2^ Menzies Institute for Medical Research, University of Tasmania, Hobart, TAS, Australia; ^3^ Department of Nuclear Medicine/PET Image Center, The Second Xiangya Hospital of Central South University, Changsha, China

**Keywords:** NSCLC, PD-L1, cost-effectiveness, pembrolizumab, squamous, non-squamous

## Abstract

**Objective** To compare the cost-effectiveness of the combination of pembrolizumab and chemotherapy (Pembro+Chemo) versus pembrolizumab monotherapy (Pembro) as the first-line treatment for metastatic non-squamous and squamous non-small-cell lung cancer (NSCLC) with PD-L1expression ≥50%, respectively, from a US health care perspective.

**Material and Methods** A comprehensive Makrov model were designed to compare the health costs and outcomes associated with first-line Pembro+Chemo and first-line Pembro over a 20-years time horizon. Health states consisted of three main states: progression-free survival (PFS), progressive disease (PD) and death, among which the PFS health state was divided into two substates: PFS while receiving first-line therapy and PFS with discontinued first-line therapy. Two scenario analyses were performed to explore satisfactory long-term survival modeling.

**Results** In base case analysis, for non-squamous NSCLC patients, Pembro+Chemo was associated with a significantly longer life expectancy [3.24 vs 2.16 quality-adjusted life-years (QALYs)] and a substantially greater healthcare cost ($341,237 vs $159,055) compared with Pembro, resulting in an ICER of $169,335/QALY; for squamous NSCLC patients, Pembro+Chemo was associated with a slightly extended life expectancy of 0.22 QALYs and a marginal incremental cost of $3,449 compared with Pembro, resulting in an ICER of $15,613/QALY. Our results were particularly sensitive to parameters that determine QALYs. The first scenario analysis yielded lower ICERs than our base case results. The second scenario analysis founded Pembro+Chemo was dominated by Pembro.

**Conclusion** For metastatic non-squamous NSCLC patients with PD-L1 expression ≥50%, first-line Pembro+Chemo was not cost-effective when compared with first-line Pembro. In contrast, for the squamous NSCLC patient population, our results supported the first-line Pembro+Chemo as a cost-effective treatment. Although there are multiple approaches that are used for extrapolating long-term survival, the optimal method has yet to be determined.

## Introduction

Lung cancer is the leading cause of cancer-related deaths in the United States and globally, contributing to roughly 25% of cancer-related deaths. Most lung cancers (∼84%) are non-small-cell lung cancer (NSCLC). During the past decade, gene therapies targeting the oncogenic drivers such as sensitizing epidermal growth factor receptor (EGFR) mutations and anaplastic lymphoma kinase (ALK) translocations, has showed a great efficacy on the management of NSCLC (Brahmer et al., 2015; Borghaei et al., 2015; Garon et al., 2015; Herbst et al., 2016; Kazandjian et al., 2016; Reck et al., 2016; Rittmeyer et al., 2017; Barlesi et al., 2018; Gandhi et al., 2018; Paz-Ares et al., 2018; Antonia et al., 2019; Mok et al., 2019; Reck et al., 2021). However, these therapies do not take effect in patients with metastatic NSCLC without driver molecular alterations, who constitutes approximately 80% of the NSCLC cases (Aisner and Marshall, 2012). This has led to a revolution in the treatment paradigm for metastatic NSCLC patients with negative targetable driver alteration. Immune-checkpoint inhibitors (ICIs), either as a monotherapy or in combination with chemotherapies, have become the backbone of the standard of care for this disease (National Comprehensive Cancer Network, 2021). Tumor cell programmed death-ligand 1 (PD-L1), as the most robust predictor of the clinical response to ICIs, is recommended to be tested to guide the selection of treatment strategies for metastatic driver-negative NSCLC (Di Federico et al., 2021; Grant et al., 2021; Pathak et al., 2021). The latest National Comprehensive Cancer Network (NCCN) guidelines for NSCLC recommend replacing traditional chemotherapies with ICIs-containing regimens as the preferred first-line therapies for NSCLC when PD-L1 expresses in at least 50% of tumor cells (National Comprehensive Cancer Network, 2021).

Pembrolizumab, used as the first-line treatment for metastatic driver-negative NSCLC with PD-L1expression ≥50%, is viewed as an important milestone in the era of immunotherapy. Precipitated by the favorable net benefits of pembrolizumab reported in the KEYNOTE-024 trial, and later, the KEYNOTE-042 trial (Reck et al., 2016; Mok et al., 2019; Reck et al., 2021), it becomes the first ICI approved by the U.S. Food and Drug Administration (FDA) used as the first-line therapy for this subset of NSCLC patients (US Food and Drug Administration, 2021). Two years later, results from the KEYNOTE-189 and KEYNOTE-407 trials found that pembrolizumab in combination with chemotherapy resulted in the higher response rate and longer survival than platinum-based chemotherapy among metastatic non-squamous NSCLC patients as well as squamous NSCLC patients, regardless of the level of PD-L1 expression. Based on this evidence, pembrolizumab +chemotherapy is recommended as a standard first-line treatment for the PD-L1-high patient population (Gandhi et al., 2018; Paz-Ares et al., 2018). However, it remains unclear whether the combination therapy is superior to the pembrolizumab monotherapy due to the lack of a decent clinical trial with head-to-head comparisons. This has posed a challenge for oncologists when making treatment decisions.

The American Cancer Society estimates that, there will be about 118, 800 new cases of metastatic NSCLC in the United States (US) in 2021 (American Cancer Society, 2021), and approximately 25–35% of these cases are expected to have high levels of tumor cell PD- L1expression (≥50%) (Sezer et al., 2021), corresponding to nearly 35,640 potential patients. ICIs-containing regimens thus represent as one of the most pressing needs in the oncology therapeutics market. Whether their excellent efficacy outweighs the financial burden they impose is the key to determine the appropriateness for their widespread use, and this emphasizes the need for economic analysis for these approved therapies. Although several US-based studies have evaluated the cost-effectiveness of pembrolizumab or pembrolizumab plus chemotherapy against platinum-based chemotherapy in the first-line treatment for this disease (Huang et al., 2017; She et al., 2019), their studies were not able to answer the comparative cost-effectiveness of using pembrolizumab alone versus using in combination with chemotherapy, and this led to an evidence-practice gap in the real-life practice. To assist in clinical decision-making, the aim of this study was to compare the cost-effectiveness of the combination of pembrolizumab and chemotherapy versus pembrolizumab monotherapy as the first-line treatment for metastatic non-squamous and squamous NSCLC with PD-L1expression ≥50% from a US health care perspective.

## Materials and Methods

### Overview

Through mathematical modeling using TreeAge Pro software (version 2021, https://www.treeage.com/) and network meta-analysis (NMA) implemented in R software (version 4.0.4, http://www.r-project.org), we compared the cost-effectiveness between first-line pembrolizumab combined chemotherapy (Pembro+Chemo) and pembrolizumab monotherapy (Pembro) indirectly among patients with metastatic non-squamous and squamous NSCLC with PD-L1 of at least 50% from a US health care perspective. This study is exempted from the institutional review board approval because it used only existing data to inform the model. Our study followed the Consolidated Health Economic Evaluation Reporting Standards (CHEERS) reporting guideline.

### Simulation Model

**FIGURE 1:**
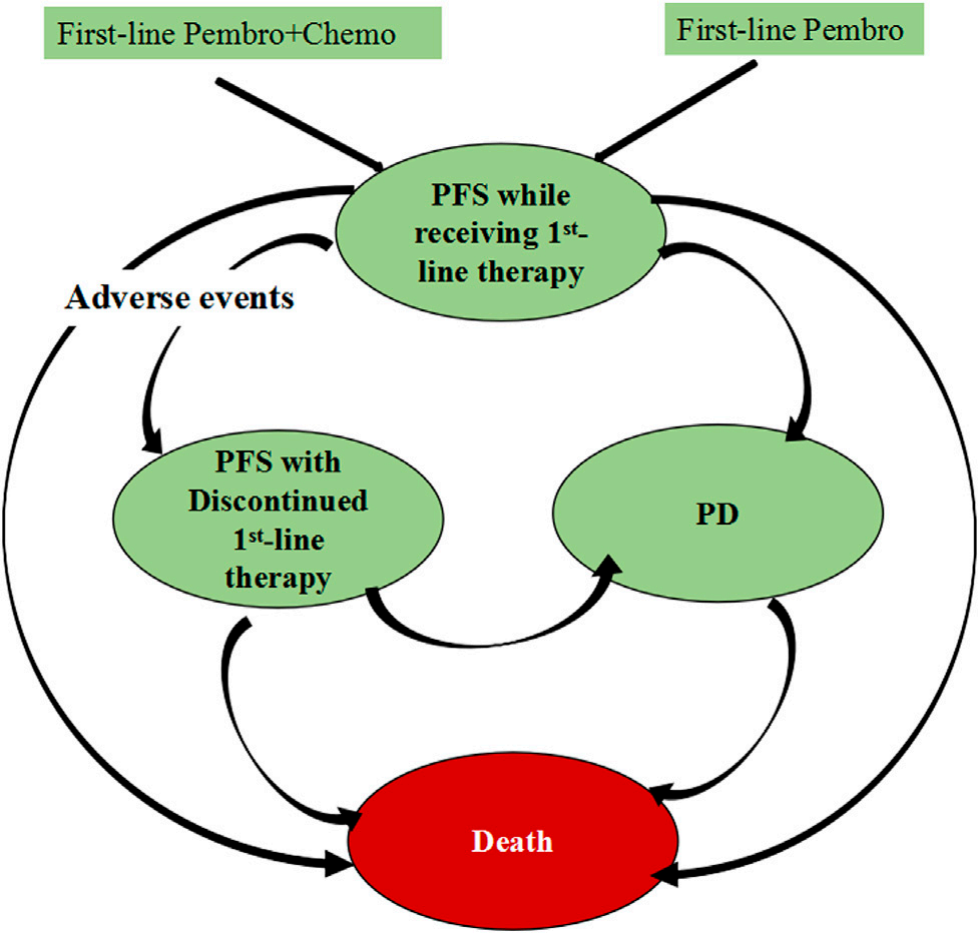
Diagram of transitions between health states. PD, progressive disease.

For this economic evaluation, we built a Markov model composed of three main health states: progression-free survival (PFS), progressive disease (PD) and death, in which PFS health state was divided into two sub-health states: progression-free survival (PFS) while receiving first-line therapy and PFS with discontinued first-line therapy (Figure 1). All patients began in the health state of PFS while receiving first-line therapy and were randomized to 2 first-line treatment strategies. Individuals who experienced intolerable toxicity during first-line treatment but did not develop disease progression could enter the health state of PFS with discontinued first-line therapy. Individuals with disease progression would enter the PD health state and receive subsequent anticancer therapy if there is sustained survival benefit, otherwise they will receive best supportive care (BSC). To better reflect the real-world practice, patients were proceeded to palliative care before death. The first-line and subsequent treatment regimens was detailed in Supplementary Table S1.

With a 1-month Markov cycle and a 20-years time horizon, the Markov model was used to project the cumulative costs and quality-adjusted life-years (QALYs) corresponding to each regimen. The incremental cost-effectiveness ratio (ICER) between two competitive regimens was then generated and compared with a willingness-to-pay (WTP) threshold of $100,000 per QALY to determine the cost-effective regimen (Neumann et al., 2014). Costs were reported in 2021 US dollars and an annual discount rate of 3% was applied for both costs and QALYs (Sanders et al., 2016).

### Transition Probabilities

The transition probabilities between three main health states were estimated by published extrapolation techniques and standard NMA techniques (Diaby et al., 2014; Guyot et al., 2012). For non-squamous and squamous NSCLC patients treated with first-line Pembro+Chemo, we ascertained overall survival (OS) and PFS rates from the Kaplan-Meier (KM) curves reported in the KEYNOTE-189 and KEYNOTE-407 trials, respectively (Gandhi et al., 2018; Paz-Ares et al., 2018). Then, the log-logistic distributions were selected to fit these recreated individual patient-level data because they yielded the lowest AIC and BIC statistics (Supplementary Table S2; Figure 1). In calculating the transition probabilities for patients receiving first-line Pembro, the hazard ratios (HRs) of first-line Pembro vs Pembro+Chemo generated by implementing NMA for non-squamous and squamous patient populations, respectively, were applied (Reck et al., 2016; Mok et al., 2019; Reck et al., 2021). Transition probabilities between the two PFS sub-health states were calculated using the clinical data regarding the discontinuation of first-line therapy owing to adverse events (AEs) (Supplementary Table S3) (Reck et al., 2016; Reck et al., 2021; Mok et al., 2019; Gandhi et al., 2018; Paz-Ares et al., 2018). Data in KYNOTE-024 trial were preferentially selected for estimating this model parameter due to its longer follow-up period (5 years) compared with the KYNOTE-042 trial (Mok et al., 2019; Reck et al., 2021). Table 1 summarizes parameters used for transition probabilities estimation.

**TABLE 1:** Model inputs.

Parameters	Baseline value	Ranges	Distribution	Source
**Survival**				
Log-logistic functions for first-line Pembro+Chemo				
OS modeling (Non-squamous NSCLC)	θ = 0.03084, *κ* = 0.92588	—	—	Estimated^a^
OS modeling (Squamous NSCLC)	θ = 0.02428, *κ* = 1.27621	—	—	Estimated^b^
PFS modeling (Non-squamous NSCLC)	θ = 0.01422, *κ* = 1.80138	—	—	Estimated^a^
PFS modeling (Squamous NSCLC)	θ = 0.00985, *κ* = 2.25359	—	—	Estimated^b^
HRs for first-line Pembro vs Pembro+Chemo				
HR_OS_ (Non-squamous NSCLC)	1.67	0.46–2.87	LogNormal	Estimated^c^
HR_OS_ (Squamous NSCLC)	1.13	0.60–2.10	LogNormal	Estimated^d^
HR_PFS_ (Non-squamous NSCLC)	1.53	0.24–2.86	LogNormal	Estimated^c^
HR_PFS_ (Squamous NSCLC)	1.06	0.14–1.81	LogNormal	Estimated^d^
1-Cycle probability of treatment discontinuation due to AEs				
Discontinuation of pembrolizumab monotherapy (Non-squamous and squamous NSCLC)	0.005559	0.002779–0.008338	Beta	Estimated^d^
Discontinuation of pembrolizumab (Non-squamous NSCLC)	0.010231	0.005115–0.015346	Beta	Estimated^d^
Discontinuation of pemetrexed (Non-squamous NSCLC)	0.011788	0.005894–0.017682	Beta	Estimated^d^
Discontinuation of platinum-based drug (Non-squamous NSCLC)	0.003613	0.001807–0.005420	Beta	Estimated^d^
Discontinuation of pembrolizumab (Squamous NSCLC)	0.011023	0.005512–0.016535	Beta	Estimated^d^
Discontinuation of paclitaxel or nab-paclitaxel (Squamous NSCLC)	0.010025	0.005013–0.015038	Beta	Estimated^d^
Discontinuation of carboplatin (Squamous NSCLC)	0.006890	0.003445–0.010336	Beta	Estimated^d^
**Costs**				
Pembrolizumab price/mg	52.75	26.38–79.13	Gamma	Centers for Medicare and Medicaid Services. (2021a)
Pemetrexed price/mg	7.49	3.75–11.24	Gamma	Centers for Medicare and Medicaid Services. (2021a)
Paclitaxel price/mg	0.13	0.07–0.20	Gamma	Centers for Medicare and Medicaid Services. (2021a)
Nab-paclitaxel price/mg	14.08	7.04–21.12	Gamma	Centers for Medicare and Medicaid Services. (2021a)
Carboplatin price/mg	0.05	0.03–0.08	Gamma	Centers for Medicare and Medicaid Services. (2021a)
Cisplatin price/mg	0.18	0.09–0.27	Gamma	Centers for Medicare and Medicaid Services. (2021a)
Chemotherapy infusion 1 h	148.30	74.15–222.45	Gamma	Centers for Medicare and Medicaid Services. (2021b)
Chemotherapy infusion additional hour	31.40	15.70–47.10	Gamma	Centers for Medicare and Medicaid Services. (2021b)
Office/Outpatient physician visit	183.19	91.60–274.79	Gamma	Centers for Medicare and Medicaid Services. (2021b)
Imaging examination	117.59	58.80–176.39	Gamma	Centers for Medicare and Medicaid Services. (2021b)
Best supportive care	637	318.50–955.50	Gamma	Criss et al. (2019)
Death associated cost	9,433	4,716.50–14,149.50	Gamma	Criss et al. (2019)
AEs management costs				
First-line Pembro (Non-squamous and squamous NSCLC)	1,400.88	700.44–2,101.31	Gamma	Estimated^e^
First-line Pembro+Chemo (Non-squamous NSCLC)	6,142.07	3,071.03–9,213.10	Gamma	Estimated^e^
First-line Pembro+Chemo (Squamous NSCLC)	5,932.63	2,966.31–8,898.94	Gamma	Estimated^e^
Subsequent anticancer therapy costs				
First-line Pembro (Non-squamous NSCLC)	12,283.00	6,141.50–18,424.50	Gamma	Insinga et al. (2018)
First-line Pembro+Chemo (Non-squamous NSCLC)	12,831.00	6,415.50–19,246.50	Gamma	Insinga et al. (2018)
First-line Pembro (Squamous NSCLC)	3,785.00	1892.50–5,677.50	Gamma	Insinga et al. (2019)
First-line Pembro+Chemo (Squamous NSCLC)	1,195.00	597.50–1792.50	Gamma	Insinga et al. (2019)
**Utilities**				
≥12 months prior to death (Non-squamous NSCLC)	0.834	0.823–0.846	Beta	Insinga et al. (2018); Garassino et al. (2020)
≥12 months prior to death (Squamous NSCLC)	0.842	0.823–0.861	Beta	Insinga et al. (2019); Mazieres et al. (2020)
6–12 months prior to death (Non-squamous NSCLC)	0.765	0.743–0.786	Beta	Insinga et al. (2018); Garassino et al. (2020)
6–12 months prior to death (Squamous NSCLC)	0.814	0.795–0.833	Beta	Insinga et al. (2019); Mazieres et al. (2020)
1–6 months prior to death (Non-squamous NSCLC)	0.709	0.690–0.728	Beta	Insinga et al. (2018); Garassino et al. (2020)
1–6 months prior to death (Squamous NSCLC)	0.737	0.717–0.756	Beta	Insinga et al. (2019); Mazieres et al. (2020)
≤1 month prior to death (Non-squamous NSCLC)	0.563	0.461–0.665	Beta	Insinga et al. (2018); Garassino et al. (2020)
≤1 month prior to death (Squamous NSCLC)	0.568	0.481–0.655	Beta	Insinga et al. (2019); Mazieres et al. (2020)
**Disutilities**				
First-line Pembro (Non-squamous and squamous NSCLC)	0.016	0.008–0.024	Beta	Estimated^e^
First-line Pembro+Chemo (Non-squamous NSCLC)	0.098	0.049–0.148	Beta	Estimated^e^
First-line Pembro+Chemo (Squamous NSCLC)	0.105	0.053–0.158	Beta	Estimated^e^
**Others**				
Body surface area (meters2)	1.79	1.78–1.80	Normal	Insinga et al. (2019); Mazieres et al. (2020)
Creatinine clearance rate (ml/min)	70	35.00–105.00	Normal	Insinga et al. (2019); Mazieres et al. (2020)
Discount rate (%)	3	0–5	Normal	Insinga et al. (2019); Mazieres et al. (2020)

NSCLC, non-small-cell lung cancer; OS, overall survival; PFS, progression-free survival; HRs, hazard ratios; AEs, adverse events.

a The log-logistic function parameters, theta (θ) and kappa (*γ*) were estimated based on survival data derived from the KEYNOTE-189 trial.

b The log-logistic function parameters, theta (θ) and kappa (*γ*) were estimated based on survival data derived from the KEYNOTE-407 trial.

c The HRs were generated using network meta-analysis based on survival data observed within the the KEYNOTE-189 and KEYNOTE-024 trials.

d The HRs were generated using network meta-analysis based on survival data observed within the the KEYNOTE-407 and KEYNOTE-024 trials.

e Estimated in Supplementary Table S5.

To explore satisfactory survival modeling, our base case analysis elected to use trial-based parametric distributions to project survival for the first 5 years, followed by the survival data from the Surveillance, Epidemiology, and End Results (SEER) database for non-squamous and squamous NSCLC patients (Supplementary Table S4) (National Cancer Institute, 2021). We also performed two scenario analyses based on other alternative methods that were used in previous cost-effectiveness studies (Wan et al., 2019; Watson et al., 2020; Patel et al., 2021). In our first scenario analysis, we applied the parametric extrapolation approach to project long-term survival for both Pembro+Chemo and Pembro arms. In our second scenario analysis, differed from estimating transition probabilities for death based on parametric distributions for the first 5 years and the SEER-observed survivals afterwards in our base case model, we combined an age-matched background mortality rate from US life tables (Supplementary Table S5) with the data regarding fatal treatment-related AEs from each clinical trial (Supplementary Table S3) for such calculations (Arias et al., 2019).

### Costs and Health Utility

Regimen related cost, AEs management costs and general treatment costs (including routine follow-up, BSC, and death-associated costs) were considered in our study and collected from a US health care perspective. The prices of first-line drugs were sourced from October 2021 Average Sales Price Drug Pricing Files available at the Centers for Medicare & Medicaid Services (CMS) (Centers for Medicare and Medicaid Services, 2021a). Acquisition of drug administration costs depended on the infusion price retrieved through the CMS Physician Fee Schedule Look-up Tool and the infusion duration requirements for each administration (Centers for Medicare and Medicaid Services, 2021b). For dosage calculation, we modeled the base case patients as having a body surface of 1.79 m^2^ and a creatinine clearance rate of 70 ml/min (Criss et al., 2019; Zhang et al., 2020), and then rounded to an integral multiple of single-use vial size to account for drug waste (Lien et al., 2016).

Costs for treating grade 3 + AEs with an incidence of at least 1% were considered in the model and were calculated as frequency-weighted averages according to the safety data reported in clinical trials (Reck et al., 2016; Gandhi et al., 2018; Paz-Ares et al., 2018). Each AE was matched to a Clinical Classification Software Refined (CCSR) diagnosis to obtain a corresponding management cost per event from the Healthcare Cost and Utilization Project (HCUP) (Supplementary Table S6) (Agency for Healthcare Research and Quality, 2021). We modeled routine follow-up as a monthly physician visit and a quarterly imaging examination and retrieved these costs from the CMS Physician Fee Schedule (Centers for Medicare and Medicaid Services, 2021b). Costs of subsequent anticancer therapy, BSC, and palliative care were obtained from the literature (Insinga et al., 2018; Criss et al., 2019; Insinga et al., 2019). All costs are outlined in Table 1.

Treatment effectiveness was measured in QALY, which was calculated as a health utilities-weighted life expectancy (overall survival). Considering that cancer patients’ health utilities varied by tumor histology, the health-related quality of life data were collected from the KEYNOTE-189 and KEYNOTE-407 trials for patients with metastatic non-squamous and squamous NSCLC, respectively (Garassino et al., 2020; Mazieres et al., 2020). A time-to-death approach described in previous studies was used to reflect the decline in quality of life as patients approach death (Insinga et al., 2018; Insinga et al., 2019). Utility decrements due to grade III/IV AEs were also considered in our model (Nafees et al., 2017). Details regarding health utilities used in the model are available in the Supplementary Table S6.

### Statistical Analysis

This Cost-effectiveness analysis was performed for patients with non-squamous and squamous NSCLC, respectively. We performed one-way deterministic sensitivity analyses (DSA) by varying individual parameters within the plausible ranges to ascertain their role in the ICER. Ranges for each parameter were modeled as the reported 95% confidence intervals (CIs), or within ±50% of the baseline value provided that its 95% CIs was not available. To further evaluate the robustness of model results, we performed a probabilistic sensitivity analysis (PSA) using Monte Carlo simulations with 10,000 iterations to determine the impact of variation in multiple parameters on the ICER. During each Monte Carlo simulation, relevant parameters were random sampled from an appropriate distribution to generate a cost and a QALY estimate. The DAS ranges and parameter distributions used in the PSA were detailed in Table 1.

## Results

### Incremental Cost-Effectiveness Ratios

The summary results of the base case analysis and scenario analyses are shown in Table 2. In our base case analysis, first-line therapy of Pembro+Chemo in metastatic non-squamous NSCLC patients was associated with a significantly longer life expectancy (3.24 vs 2.16 QALYs) and a substantially greater healthcare cost ($341,237 vs $159,055) compared with first-line Pembro, producing an ICER of $169,335 per QALY above the WTP threshold of $100,000 per QALY. For metastatic squamous NSCLC patients, first-line therapy of Pembro+Chemo was associated with a slightly extended life expectancy of 0.22 QALYs and a marginal incremental cost of $3,449 compared with first-line Pembro, generating an ICER of $15,613 per QALY below the WTP threshold used in the model.

**TABLE 2:** Summary results.

Results	Cost,$	QALYs			Incremental		ICER, $/QALY
		PFS	PD	OS	Cost,$	QALYs	
**Non-squamous NSCLC population**							
Base case analysis							
Pembrolizumab	159,055	0.69	1.47	2.16	—	—	—
Pembrolizumab+chemotherapy	341,237	1.08	2.16	3.24	182,182	1.08	169,335
First scenario analysis							
Pembrolizumab	163,546	0.69	1.86	2.55	—	—	—
Pembrolizumab+chemotherapy	363,726	1.11	3.74	4.85	200,181	2.30	86,990
Second scenario analysis							
Pembrolizumab	172,605	0.72	2.53	3.25	—	—	—
Pembrolizumab+chemotherapy	342,253	1.08	2.05	3.13	169,647	−0.11	Dominated
**Squamous NSCLC population**							
Base case analysis							
Pembrolizumab	150,444	0.67	1.01	1.68	—	—	—
Pembrolizumab+chemotherapy	153,892	0.71	1.19	1.90	3,449	0.22	15,613
First scenario analysis							
Pembrolizumab	152,428	0.67	1.18	1.85	—	—	—
Pembrolizumab+chemotherapy	157,332	0.71	1.49	2.20	4,904	0.35	13,956
Second scenario analysis							
Pembrolizumab	166,607	0.69	2.60	3.29	—	—	—
Pembrolizumab+chemotherapy	167,647	0.73	2.44	3.17	1,040	−0.13	Dominated

In our first scenario analysis, the increases in QALYs associated with first-line Pembro+Chemo were more significant than the increase in cost, resulting in relatively lower ICERs than our base case results ($86,990/QALY vs $169,335/QALY for non-squamous NSCLC patient and $13,956/QALY vs $15,613/QALY for squamous NSCLC patient, respectively). In our second scenario analysis, first-line Pembro+Chemo was associated with lower QALYs and higher costs when compared with first-line Pembro, resulting in first-line Pembro+Chemo being dominated by first-line Pembro.

### Sensitivity Analysis

**FIGURE 2:**
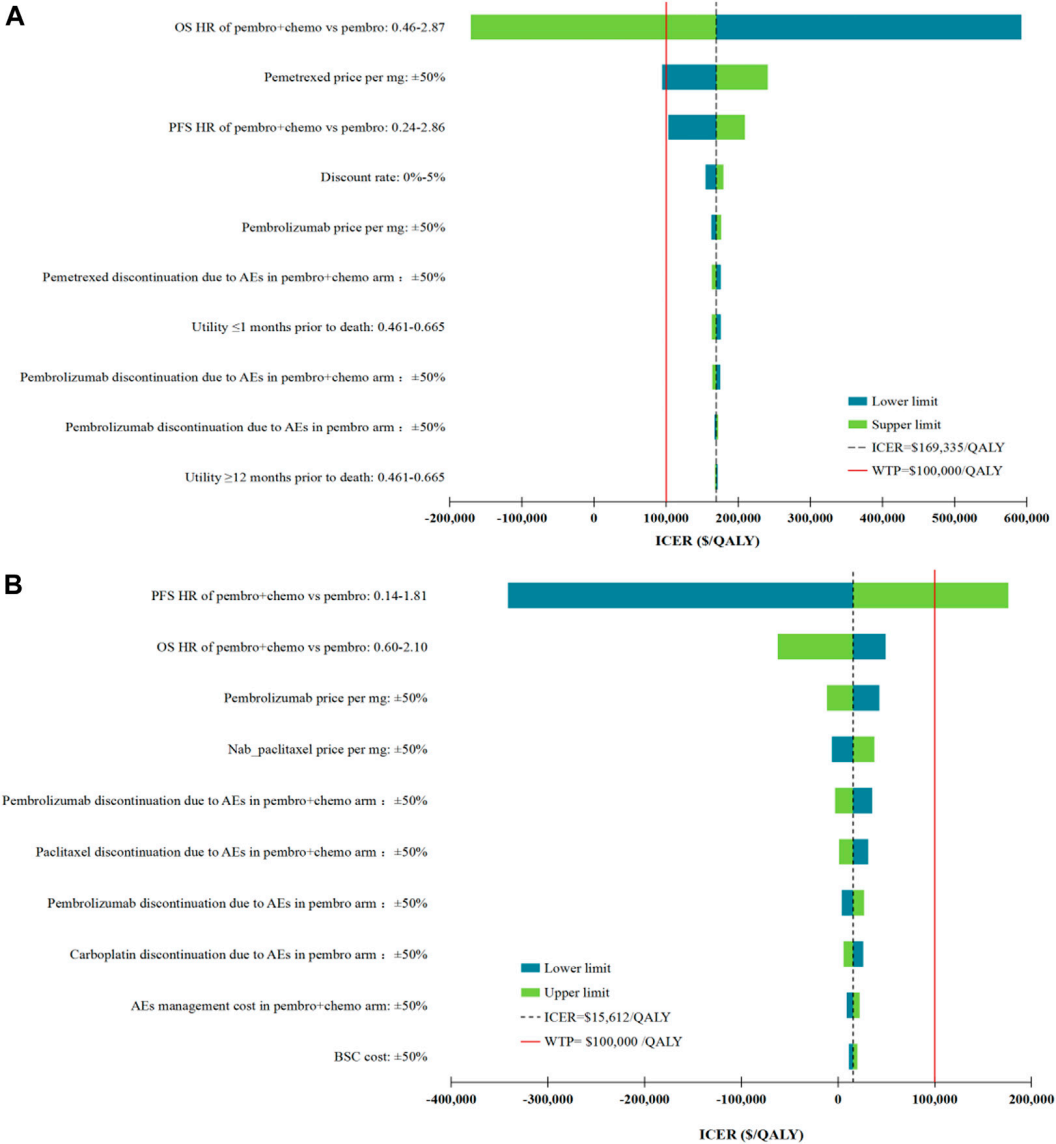
Deterministic sensitivity analysis results. **(A)**, the top 10 parameters with the greatest influence on the ICER of first-line Pembro+Chemo vs Pembro in non-squamous NSCLC patient population; **(B)**, the top 10 parameters with the greatest influence on the ICER of first-line Pembro+Chemo vs Pembro in squamous NSCLC patient population. ICER, incremental cost-effectiveness ratios; QALY, quality-adjusted life-years; AEs, adverse events; OS, overall survival; PFS, progression-free survival; HR, hazard ratios; BSC, best supportive care.

DSA results of our base case analysis for non-squamous NSCLC patient population showed that only the fluctuations in the OS HR for first-line Pembro+Chemo relative to Pembro, and pemetrexed price/mg had the potential to make first-line Pembro+Chemo cost-effective compared with first-line Pembro. Meanwhile, the lower limit of PFS HR of Pembro+Chemo vs Pembro led to the ICER approaching the WTP threshold of $100,000 per QALY ($102,964 per QALY). Other parameters had minimal effects on our model results (the ICERs ranged between $154,536/QALY and $179,304/QALY). When DSA was performed in squamous NSCLC patient population, the ICERs of first-line Pembro+Chemo vs Pembro remained below the WTP threshold at the lower or upper limits of any tested parameter except for PFS HR for Pembro+Chemo vs Pembro. The tornado diagram in Figure 2 shows the DSA results.

The PSA results of the base case revealed that, at a WTP threshold of $100,000 per QALY, the probability of first-line Pembro+Chemo being cost-effective in non-squamous and squamous NSCLC patient populations were 3.6 and 87.4%, respectively (Supplementary Figure S2). When we increased the WTP threshold, an expected increase in the cost-effectiveness probability of first-line Pembro+Chemo was observed.

## Discussion

Through mathematical modeling and NMA, we evaluated the cost-effectiveness of first-line Pembro+Chemo relative to first-line Pembro among metastatic NSCLC patients with PD-L1 expression ≥50% from the US health care sector perspective. In our base case analysis, we found that in non-squamous NSCLC patient population, first-line Pembro+Chemo was superior to first-line Pembro in survival, but was associated with an overwhelming healthcare cost. Since the reported ICER ($169,335 per QALY) exceeded the WTP threshold of $100,000 per QALY used in the current study, first-line Pembro+Chemo was not cost-effective compared with first-line Pembro. In contrast, for the squamous NSCLC patient population, our results support the first-line use of Pembro+Chemo as a cost-effective treatment by showing that first-line Pembro+Chemo added a 0.22 QALYs at a marginal incremental cost of $3,449 and the generated ICER ($15,613 per QALY) was far below the WTP threshold.

Among the top 10 most sensitive parameters, the parameters that determine QALY were superior in numbers, including HRs of first-line Pembro+Chemo vs Pembro, first-line drug discontinuation due to AEs and health utilities (Figure 2). Of note, these parameters, as quantitative indicators reflecting the efficacy and safety of cancer treatment, were difficult to be changed through clinical or policy interventions. Apart from these QALY drivers, drug prices also had considerable influences on our cost-effectiveness results, raising concerns regarding the role of drug cost in determining the preferred regimen. Combining this findings with the findings in our previous cost-effectiveness studies (Liu et al., 2020a; Liu et al., 2021), we found that the cost variance between two competing treatments has the most potential to reverse the results of cost-effectiveness analysis. DSA confirmed that among all drugs, the result of non-squamous NSCLC patient population was most affected by the pemetrexed price/mg, because pemetrexed was a supplement to first-line Pembro+Chemo compared with first-line Pembro, with maintenance treatment costs of $ 6,700 per 3-weeks. For squamous NSCLC patient population, the pembrolizumab price/mg ranked the first in the DSA among all drugs, mainly due to the difference in the probabilities of first-line pembrolizumab discontinuation due to AEs.

Since cost-effectiveness evaluation focuses on whether a new treatment can prolong life expectancy (overall survival) at an affordable cost (Ferguson et al., 2000), we pay more attention to the estimation of overall survival in the current study. From the existing literature, parameter fitting method alone or combined with SEER-observed survivals, as well as background mortality rate application technology, are the three mainstream methods to estimate overall survivals in cost-effectiveness research (Criss et al., 2019; Wan et al., 2019; Watson et al., 2020; Zhang et al., 2020; Patel et al., 2021). To better understand the applicability of these methods, two scenario analyses were conducted in the present study. In the first scenario analysis, we found that the best fitting parametric projection increased discounted life expectancy relative to SEER population data. Our previous studies have pointed out that extrapolating long-term survival from the trial-based parameter distribution inevitably suffers from uncertainty (Wan et al., 2019; Liu et al., 2020b). SEER-observed survival data may be more applicable as the data reflect the real-world performance. In the second scenario analysis, when we combined an age-matched background mortality rate with the clinical data of fatal treatment-related AEs to calculate the transition probabilities for death, the results showed that first-line Pembro+Chemo gained lower QALYs than first-line Pembro. However, first-line Pembro+Chemo is generally known to be superior to first-line Pembro in survival (Di Federico et al., 2021; Pathak et al., 2021), so this result is likely to be untenable. Therefore, background mortality rate application technology should be used with caution because it lacks the ability to directly map clinical effects of treatments.

To our knowledge, there are two existing studies from the same authors (Insinga et al.) on the cost-effectiveness of first-line Pembro+Chemo vs first-line Pembro for the US non-squamous and squamous NSCLC patient population, respectively (Insinga et al., 2018; Insinga et al., 2019). The study of non-squamous NSCLC with PD-L1 expression≥50% reported a slightly lower ICER for first-line Pembro+Chemo vs first-line Pembro ($147,365 per QALY vs $169,335 QALY, respectively) (Insinga et al., 2018). The authors cited a WTP threshold of 3 times per capita gross domestic product (GDP) ($180,000/QALY) and concluded that the first-line Pembro+Chemo was cost-effective among this patient population. Another cost-effectiveness study found that first-line Pembro+Chemo was associated with reduced net cost and improved QALYs, and therefore a cost-saving strategy (Insinga et al., 2019). Some inconsistency between our findings and the findings in the above two studies can be explained by the facts that the current model considered the first-line treatment discontinuations due to AEs and the utility decrements due to AEs, and the current study applied NMA to perform the indirect cost-effectiveness comparison between first-line Pembro+Chemo and first-line Pembro while the above studies used Bucher method.

This study has several notable strengths. First, we utilized the most detailed clinical efficacy and safety data regarding first-line Pembro+Chemo and Pembro to describe the cost-effectiveness of these two widely used and controversial treatments for non-squamous and squamous NSCLC patients with PD-L1 expression ≥50%, respectively. Therefore, our analysis has provided valuable evidence for clinicians to make relevant decisions on treatments. Second, we employed three mainstream survival modeling methods to build the cost-effectiveness model and analyzed the corresponding results to judge their applicability to the current research. This study is the first to examine the applicability of the three methods and therefore have implications for survival modeling in future economic evaluation. Third, our model comprehensively considers the impact of AEs, including the first-line treatment discontinuations due to AEs, as well as the incidences, costs and disutility associated with grade III/IV AEs.

This study also has several limitations. First, there is inherent uncertainty in the costs used to populate the model. However, a series of sensitivity analyses found that our results are not particularly sensitive to cost parameters, indicating that including more accurate estimates is unlikely to change our results. Second, we modeled proportions of patients receiving subsequent anticancer therapy based on clinical trial data, which may not reflect the prevalence of subsequent anticancer therapy used in real-world practice. Third, our model did not include traditional chemotherapy because ICI-containing regimens have replaced traditional chemotherapy as the standard first-line therapy for metastatic NSCLC with PD-L1 expression≥50%. Fourth, the treatments analyzed in this trial-based economic assessment may not fully reflect the real-world performance. More evidence from real-life scenarios is needed to be collected to verify our results.

## Conclusion

This economic evaluation found that for metastatic non-squamous NSCLC patients with PD-L1expression ≥50%, first-line Pembro+Chemo was not cost-effective when compared with first-line Pembro. In contrast, for the squamous NSCLC patient population, our results support the first-line Pembro+Chemo as a cost-effective treatment. Although there are multiple approaches that are used for extrapolating long-term survival, the optimal method has yet to be determined.

## Data Availability

The original contributions presented in the study are included in the article/Supplementary Material, further inquiries can be directed to the corresponding authors.

## Supplementary Material

Supplementary Figure S1 Model Validation for first-line Pembro+Chemo. NSCLC, non-small-cell lung cancer; OS, overall survival; PFS, progression-free survival.

Supplementary Figure S2 The probability of first-line Pembro+Chemo being cost-effective against first-line Pembro under different WTP thresholds in the non-squamous and squamous NSCLC patient populations. QALY, quality-adjusted life-year; NSCLC, non-small-cell lung cancer.
