# Supplementary material for: Cost-Effectiveness of Pembrolizumab Plus Chemotherapy Versus Pembrolizumab Monotherapy in Metastatic Non-Squamous and Squamous NSCLC Patients With PD-L1 Expression ≥ 50%
Source: Front Pharmacol. 2022 Jan 10;12:803626. doi: 10.3389/fphar.2021.803626 (PMC8784520; doi:10.3389/fphar.2021.803626)
Supplement: Supplementary file 1 [file Table1.DOCX]

Table 1. Treatment regimens included in the model.

| **Histology type** | **Regimen** | **Dosage** | **Schedule** | **Injection duration** | **% Receiving subsequent anticancer therapy in discontinued patients** |
| --- | --- | --- | --- | --- | --- |
| Non-squamous NSCLC | First-line Pembro | Pembrolizumab, 200 mg fixed | every 3-week for up to 35 cycles | More than 30 minutes | 31.0% |
|  | First-line  Pembro+Chemo | Pembrolizumab, 200 mg fixed | every 3-week for up to 35 cycles | More than 30 minutes | 45.8% |
|  |  | Pemetrexed, 500 mg/m^2^ | every 3-week | More than 10 minutes |  |
|  |  | Cisplatin,75 mg/m^2^ or carboplatin, AUC 5.0 mg/ml/min | every 3-week for 4 cycles | 30 minutes to 2 hours; 15 to 60 minutes |  |
| Squamous NSCLC | First-line Pembro | Pembrolizumab, 200 mg fixed | every 3-week for up to 35 cycles | More than 30 minutes | 31.0% |
|  | First-line  Pembro+Chemo | pembrolizumab, 200 mg fixed | every 3-week for up to 35 cycles | More than 30 minutes | 27.4% |
|  |  | Paclitaxel, 200 mg/m^2^ or nab-paclitaxel, 300 mg/m^2^ | every 3-week for 4 cycles | 3 hours; 30 minutes |  |
|  |  | Carboplatin, AUC 6.0 mg/ml/min | every 3-week for 4 cycles | 15 to 60 minutes |  |

*NSCLC, non-small-cell lung cancer; AUC, area under the concentration-time curve.*
