# Supplementary figures and images for: Cost-Effectiveness of Pembrolizumab Plus Chemotherapy Versus Pembrolizumab Monotherapy in Metastatic Non-Squamous and Squamous NSCLC Patients With PD-L1 Expression ≥ 50%
Source: Front Pharmacol. 2022 Jan 10;12:803626. doi: 10.3389/fphar.2021.803626 (PMC8784520; doi:10.3389/fphar.2021.803626)

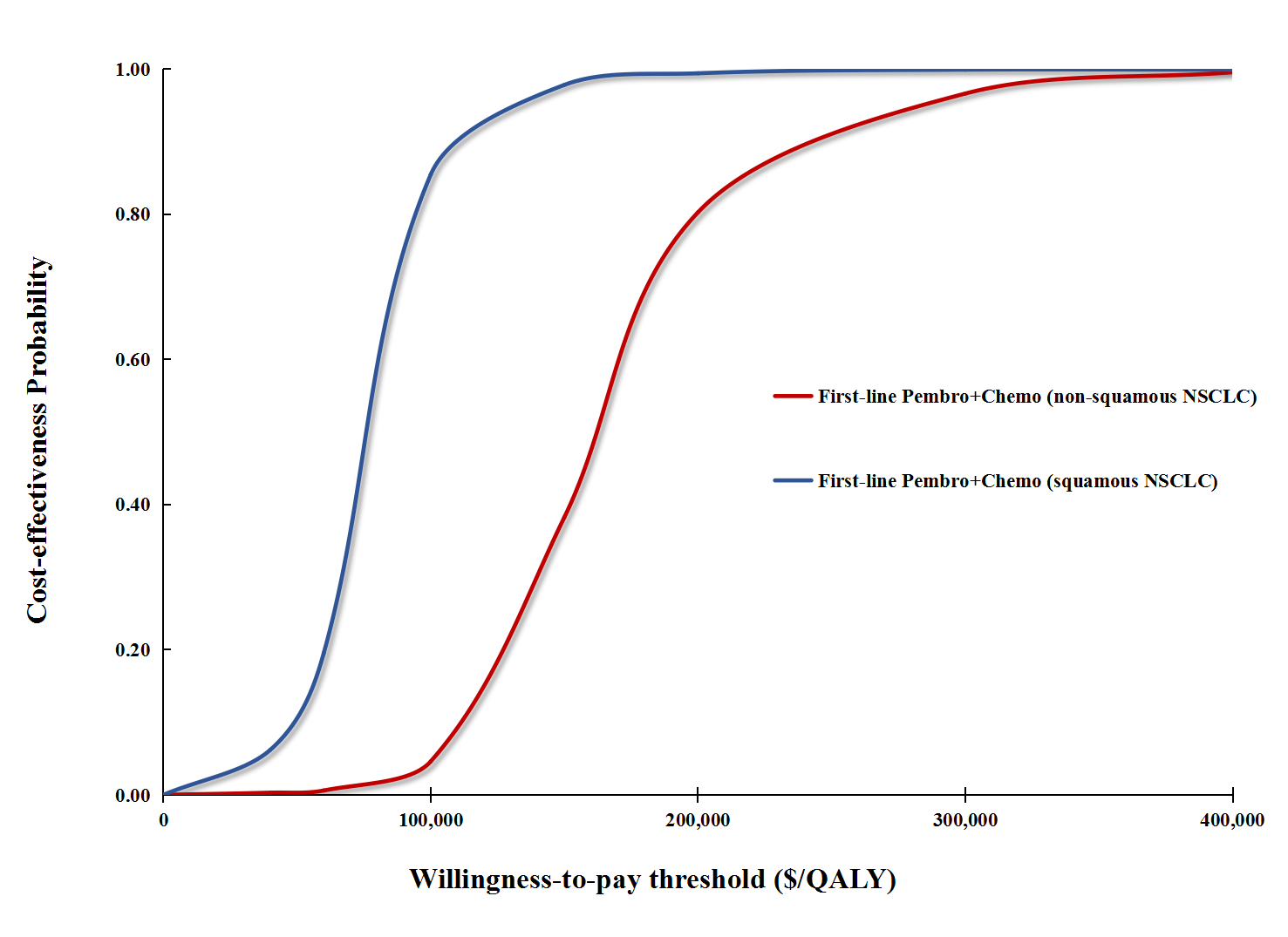

Supplement: Supplementary file 2 [file Image2.TIF]

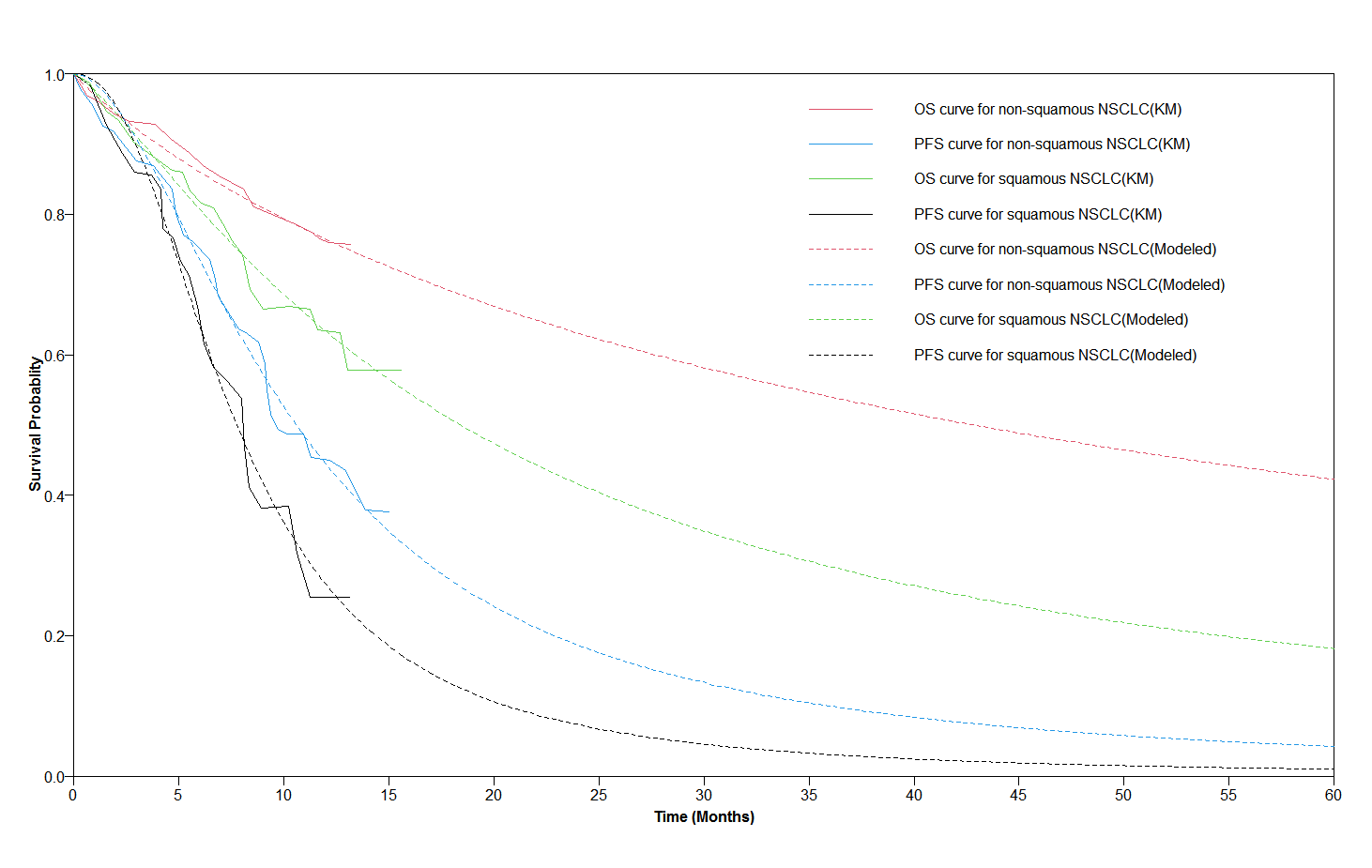

Supplement: Supplementary file 3 [file Image1.TIF]
