# Supplementary material for: Cost-Effectiveness of Pembrolizumab Plus Chemotherapy Versus Pembrolizumab Monotherapy in Metastatic Non-Squamous and Squamous NSCLC Patients With PD-L1 Expression ≥ 50%
Source: Front Pharmacol. 2022 Jan 10;12:803626. doi: 10.3389/fphar.2021.803626 (PMC8784520; doi:10.3389/fphar.2021.803626)
Supplement: Supplementary file 4 [file Table2.DOCX]

Table 2. AIC and BIC statistics of alternate parametric distributions for first-line pembrolizumab+chemotherapy

| **Histology type**  **Parametric distribution** | **Non-squamous** | | | | **Squamous** | | | |
| --- | --- | --- | --- | --- | --- | --- | --- | --- |
|  | **OS data** | | **PFS data** | | **OS data** | | **PFS data** | |
|  | **AIC** | **BIC** | **AIC** | **BIC** | **AIC** | **BIC** | **AIC** | **BIC** |
| Exponential | -103.705 | -101.434 | -108.473 | -105.251 | -179.522 | -176.095 | -51.575 | -49.219 |
| Weibull | -141.321 | -136.832 | -137.072 | -132.240 | -91.694 | -88.038 | -83.897 | -80.363 |
| Lognormal | -112.534 | -109.127 | -133.887 | -129.055 | -181.970 | -186.829 | -89.393 | -85.859 |
| Loglogistic | -171.892 | -168.486 | -157.037 | -152.204 | -199.720 | -194.579 | -93.920 | -90.386 |

*OS, overall survival; PFS, progression-free survival; AIC, Akaike information criterion; BIC, Bayesian information criterion.*
