# Supplementary material for: Cost-Effectiveness of Pembrolizumab Plus Chemotherapy Versus Pembrolizumab Monotherapy in Metastatic Non-Squamous and Squamous NSCLC Patients With PD-L1 Expression ≥ 50%
Source: Front Pharmacol. 2022 Jan 10;12:803626. doi: 10.3389/fphar.2021.803626 (PMC8784520; doi:10.3389/fphar.2021.803626)
Supplement: Supplementary file 5 [file Table5.DOCX]

Table 5. Age-matched background mortality rate from US life tables

| **Age (years)** | **Probability of dying between ages x and x + 1** | **Age (years)** | **Probability of dying between ages x and x + 1** |
| --- | --- | --- | --- |
| 63-64 | 0.011181 | 82-83 | 0.059240 |
| 64-65 | 0.011922 | 83-84 | 0.066564 |
| 65-66 | 0.012710 | 84-85 | 0.074045 |
| 66-67 | 0.013621 | 85-86 | 0.081954 |
| 67-68 | 0.014620 | 86-87 | 0.090879 |
| 68-69 | 0.015770 | 87-88 | 0.101938 |
| 69-70 | 0.017100 | 88-89 | 0.114075 |
| 70-71 | 0.018428 | 89-90 | 0.127331 |
| 71-72 | 0.020317 | 90-91 | 0.141733 |
| 72-73 | 0.022102 | 91-92 | 0.157289 |
| 73-74 | 0.024194 | 92-93 | 0.173986 |
| 74-75 | 0.026342 | 93-94 | 0.191788 |
| 75-76 | 0.029042 | 94-95 | 0.210633 |
| 76-77 | 0.032001 | 95-96 | 0.230432 |
| 77-78 | 0.035443 | 96-97 | 0.251066 |
| 78-79 | 0.039257 | 97-98 | 0.272395 |
| 79-80 | 0.043393 | 98-99 | 0.294253 |
| 80-81 | 0.048163 | 99-100 | 0.316456 |
| 81-82 | 0.053216 | 100 and over | 1.000000 |
