# Supplementary material for: Cost-Effectiveness of Pembrolizumab Plus Chemotherapy Versus Pembrolizumab Monotherapy in Metastatic Non-Squamous and Squamous NSCLC Patients With PD-L1 Expression ≥ 50%
Source: Front Pharmacol. 2022 Jan 10;12:803626. doi: 10.3389/fphar.2021.803626 (PMC8784520; doi:10.3389/fphar.2021.803626)
Supplement: Supplementary file 6 [file Table3.DOCX]

Table 3. Proportion and 1-cyle probability of first-line treatment discontinuation and death due to AEs.

| **First-line Regimen** | **Number of patients**  **(probability)** | **Median OS**  **(months)** | **Instantaneous rate** | **1-cyle probability** |
| --- | --- | --- | --- | --- |
| Pembrolizumab monotherapy (KEYNOTE-024) (N=154) |  |  |  |  |
| Discontinuation of pembrolizumab due to AEs | 21 (0.136364) | 26.3 | 0.005574 | 0.005559 |
| Death due to AEs | 2(0.012987) | 26.3 | 0.000497 | 0.000497 |
| Pembrolizumab+chemotherapy (KEYNOTE-189) (N=405) |  |  |  |  |
| Discontinuation of pembrolizumab due to AEs | 82 (0.202469) | 22.0 | 0.010283 | 0.010231 |
| Discontinuation of pemetrexed due to AEs | 93 (0.229630) | 22.0 | 0.011858 | 0.011788 |
| Discontinuation of platinum-based drug due to AEs | 31(0.076543) | 22.0 | 0.003620 | 0.003613 |
| Death due to AEs | 29(0.071605) | 22.0 | 0.003377 | 0.003371 |
| Pembrolizumab+chemotherapy (KEYNOTE-407) (N=278) |  |  |  |  |
| Discontinuation of pembrolizumab due to AEs | 48 (0.172662) | 17.1 | 0.011084 | 0.011023 |
| Discontinuation of carboplatin due to AEs | 31 (0.111511) | 17.1 | 0.006914 | 0.006890 |
| Discontinuation of paclitaxel or nab-paclitaxel due to AEs | 44 (0.158273) | 17.1 | 0.010076 | 0.010025 |
| Death due to AEs | 23(0.082734) | 17.1 | 0.005050 | 0.005037 |

*The following formula was applied to convert the probabilities of AEs-related treatment discontinuation during a clinical trial period into a 1-cylce probability of the events:* $p=1-exp(-rt)$*, where p indicates the probability, r is the instantaneous rate and t is the time period. OS, overall survival; AEs, adverse events.*
