# Supplementary material for: Cost-Effectiveness of Pembrolizumab Plus Chemotherapy Versus Pembrolizumab Monotherapy in Metastatic Non-Squamous and Squamous NSCLC Patients With PD-L1 Expression ≥ 50%
Source: Front Pharmacol. 2022 Jan 10;12:803626. doi: 10.3389/fphar.2021.803626 (PMC8784520; doi:10.3389/fphar.2021.803626)
Supplement: Supplementary file 7 [file Table4.DOCX]

Table 4. SEER survival statistic: advanced NSCLC (2000-2018).

| **Months** | **Survival Estimates** | | **Months** | **Survival Estimates** | | **Months** | **Survival Estimates** | |
| --- | --- | --- | --- | --- | --- | --- | --- | --- |
|  | **Squamous** | **Non-Squamous** |  | **Squamous** | **Non-Squamous** |  | **Squamous** | **Non-Squamous** |
| 61 | 4.39% | 3.28% | 101 | 2.27% | 1.83% | 141 | 1.33% | 1.16% |
| 62 | 4.29% | 3.23% | 102 | 2.27% | 1.79% | 142 | 1.29% | 1.14% |
| 63 | 4.23% | 3.18% | 103 | 2.24% | 1.76% | 143 | 1.27% | 1.14% |
| 64 | 4.15% | 3.13% | 104 | 2.20% | 1.72% | 144 | 1.26% | 1.13% |
| 65 | 4.09% | 3.07% | 105 | 2.15% | 1.71% | 145 | 1.24% | 1.12% |
| 66 | 4.02% | 2.99% | 106 | 2.12% | 1.71% | 146 | 1.23% | 1.12% |
| 67 | 3.92% | 2.94% | 107 | 2.07% | 1.70% | 147 | 1.21% | 1.12% |
| 68 | 3.87% | 2.90% | 108 | 2.06% | 1.68% | 148 | 1.17% | 1.12% |
| 69 | 3.81% | 2.84% | 109 | 2.02% | 1.65% | 149 | 1.16% | 1.12% |
| 70 | 3.76% | 2.80% | 110 | 1.99% | 1.64% | 150 | 1.16% | 1.08% |
| 71 | 3.70% | 2.76% | 111 | 1.94% | 1.62% | 151 | 1.10% | 1.08% |
| 72 | 3.64% | 2.71% | 112 | 1.92% | 1.59% | 152 | 1.08% | 1.06% |
| 73 | 3.58% | 2.65% | 113 | 1.91% | 1.57% | 153 | 1.06% | 1.03% |
| 74 | 3.50% | 2.61% | 114 | 1.84% | 1.57% | 154 | 1.04% | 1.02% |
| 75 | 3.43% | 2.59% | 115 | 1.83% | 1.56% | 155 | 1.04% | 1.02% |
| 76 | 3.37% | 2.55% | 116 | 1.81% | 1.53% | 156 | 1.01% | 1.02% |
| 77 | 3.31% | 2.51% | 117 | 1.81% | 1.50% | 157 | 0.99% | 1.02% |
| 78 | 3.26% | 2.45% | 118 | 1.78% | 1.49% | 158 | 0.97% | 1.02% |
| 79 | 3.18% | 2.42% | 119 | 1.78% | 1.47% | 159 | 0.94% | 1.02% |
| 80 | 3.14% | 2.39% | 120 | 1.77% | 1.46% | 160 | 0.94% | 1.00% |
| 81 | 3.07% | 2.33% | 121 | 1.75% | 1.45% | 161 | 0.91% | 0.98% |
| 82 | 3.01% | 2.29% | 122 | 1.72% | 1.44% | 162 | 0.91% | 0.98% |
| 83 | 2.97% | 2.25% | 123 | 1.69% | 1.42% | 163 | 0.91% | 0.98% |
| 84 | 2.93% | 2.23% | 124 | 1.68% | 1.40% | 164 | 0.91% | 0.96% |
| 85 | 2.85% | 2.19% | 125 | 1.64% | 1.37% | 165 | 0.91% | 0.94% |
| 86 | 2.82% | 2.18% | 126 | 1.63% | 1.37% | 166 | 0.88% | 0.94% |
| 87 | 2.76% | 2.17% | 127 | 1.62% | 1.35% | 167 | 0.84% | 0.94% |
| 88 | 2.71% | 2.13% | 128 | 1.60% | 1.32% | 168 | 0.79% | 0.94% |
| 89 | 2.66% | 2.10% | 129 | 1.55% | 1.31% | 169 | 0.79% | 0.94% |
| 90 | 2.62% | 2.07% | 130 | 1.55% | 1.30% | 170 | 0.79% | 0.91% |
| 91 | 2.58% | 2.05% | 131 | 1.51% | 1.28% | 171 | 0.79% | 0.91% |
| 92 | 2.55% | 2.02% | 132 | 1.45% | 1.26% | 172 | 0.79% | 0.91% |
| 93 | 2.51% | 1.98% | 133 | 1.42% | 1.23% | 173 | 0.79% | 0.91% |
| 94 | 2.49% | 1.95% | 134 | 1.42% | 1.23% | 174 | 0.79% | 0.91% |
| 95 | 2.47% | 1.93% | 135 | 1.40% | 1.23% | 175 | 0.79% | 0.80% |
| 96 | 2.45% | 1.90% | 136 | 1.38% | 1.21% | 176 | 0.79% | 0.80% |
| 97 | 2.39% | 1.89% | 137 | 1.35% | 1.20% | 177 | 0.79% | 0.80% |
| 98 | 2.35% | 1.86% | 138 | 1.35% | 1.19% | 178 | 0.79% | 0.80% |
| 99 | 2.32% | 1.86% | 139 | 1.33% | 1.18% | 179 | 0.79% | 0.57% |
| 100 | 2.29% | 1.84% | 140 | 1.33% | 1.18% | 180 | + | + |

*SEER, Surveillance, Epidemiology, and End Results; NSCLC, Non-small cell lung cancer.*
