# Supplementary material for: Cost-Effectiveness of Pembrolizumab Plus Chemotherapy Versus Pembrolizumab Monotherapy in Metastatic Non-Squamous and Squamous NSCLC Patients With PD-L1 Expression ≥ 50%
Source: Front Pharmacol. 2022 Jan 10;12:803626. doi: 10.3389/fphar.2021.803626 (PMC8784520; doi:10.3389/fphar.2021.803626)
Supplement: Supplementary file 8 [file Table6.DOCX]

Table 6. Incidences, costs and disutilities of grade III/IV AEs considered in the model.

| AEs | Proportion (%) | | | Cost per event ($) | Disutility |
| --- | --- | --- | --- | --- | --- |
|  | Pembro (KEYNOTE-024) | Pembro+Chemo (KEYNOTE-189) | Pembro+Chemo  (KEYNOTE-407) |  |  |
| Anemia | 1.30% | 18.27% | 15.83% | 6236 | /^b^ |
| Nausea | /^a^ | 3.46% | 1.44% | 5318 | /^b^ |
| Decreased appetite | /^a^ | 1.23% | 2.52% | 7915 | /^b^ |
| Peripheral neuropathy | /^a^ | /^a^ | 1.08% | 6616 | /^b^ |
| Arthralgia | /^a^ | /^a^ | 2.16% | 5553 | /^b^ |
| Dyspnea | /^a^ | 4.20% | 1.80% | 13205 | /^b^ |
| Asthenia | /^a^ | 6.67% | 2.16% | 978 | /^b^ |
| Thrombocytopenia | /^a^ | 8.40% | 8.27% | 9569 | /^b^ |
| Back pain | /^a^ | 1.48% | /^a^ | 5553 | /^b^ |
| Pneumonia | 2.60% | 2.96% | 3.24% | 7301 | /^b^ |
| Severe skin reaction | 5.19% | 2.22% | 1.44% | 6749 | /^b^ |
| Colitis | 1.95% | 1.48% | 2.52% | 7763 | /^b^ |
| Nephritis | 0.65% | 1.48% | 2.16% | 8202 | /^b^ |
| Infusion reactions | 0.65% | 0.25% | 1.80% | 4447 | /^b^ |
| Neutropenia | /^a^ | 16.05% | 23.02% | 8026 | 0.35 |
| Fatigue | 1.95% | 6.91% | 4.68% | 8397 | 0.29 |
| Diarrhoea | 3.90% | 5.19% | 4.32% | 7915 | 0.22 |
| Vomiting | /^a^ | 3.95% | 0.36% | 5318 | 0.20 |
| Rash | 1.30% | 1.98% | 0.72% | 5724 | 0.15 |
| **Estimated AEs costs and disutility** | | | |  |  |
| AEs cost for first-line Pembro (KEYNOTE-024), $ | | | | 1400.88 |  |
| AEs cost for first-line Pembro+Chemo (KEYNOTE-189), $ | | | | 6044.35 |  |
| AEs cost for first-line Pembro+Chemo (KEYNOTE-407), $ | | | | 5733.33 |  |
| AEs disutility for first-line Pembro (KEYNOTE-024) | | | |  | 0.016 |
| AEs disutility for first-line Pembro+Chemo (KEYNOTE-189) | | | |  | 0.098 |
| AEs disutility for first-line Pembro+Chemo (KEYNOTE-407) | | | |  | 0.105 |

*AEs, adverse events.*

*^a^These AEs were not observed in the KEYNOTE-024 clinical trial.*

*^b^The utility decrements regarding these AEs were not reported.*
